# Bis­(ethanol-κ*O*)bis(1-ferrocenyl-4,4,4-tri­fluoro­butane-1,3-dionato-κ^2^ *O*,*O*′)nickel(II)

**DOI:** 10.1107/S2414314621006933

**Published:** 2021-07-13

**Authors:** Wenqing Su, Tianyue Fu, Zhongwei Xu

**Affiliations:** a Department of Chemistry, Anhui University, Hefei, Anhui 230039, People’s Republic of China; Benemérita Universidad Autónoma de Puebla, México

**Keywords:** crystal structure, ferrocene, nickel

## Abstract

In the title compound, [NiFe_2_(C_5_H_5_)(C_9_H_5_F_3_O_2_)_2_(C_2_H_6_O)_2_], the central Ni^II^ ion is observed in an octa­hedral coordination environment. The chelating *β*-diketonate ligands are substituted by ferrocene, a lipophilic organometallic moiety. The ferrocene groups have the normal geometry, with eclipsed cyclo­penta­diene rings. Coordinated ethanol mol­ecules are engaged in inter­molecular hydrogen bonds, and the crystal is further stabilized by weak C—H⋯F and C—H⋯π contacts.

## Abstract 2

In this novel trimetallic complex, the central Ni^II^ ion is six-coordinate, forming an octa­hedral [NiO_6_] core.

## Graphical abstract

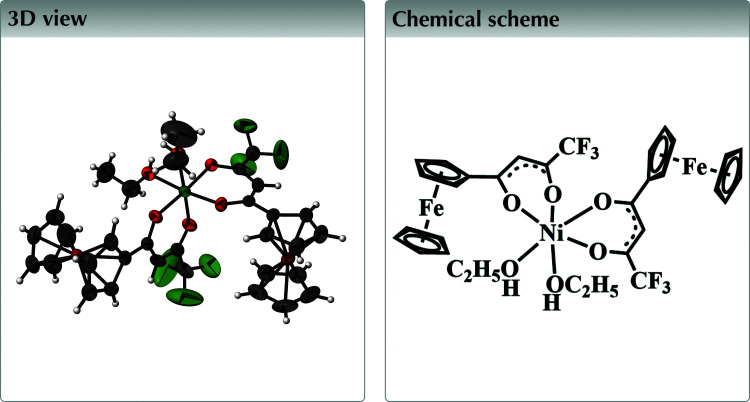

## Structure description

The introduction of the lipophilic organometallic moiety ferrocene, a compound with a sandwich-like structure, in an existing bioactive mol­ecule, is a promising tool for the development of new more efficient drugs with innovative mechanisms of action (Ludwig *et al.*, 2019 ▸). As a result of their lipophilic character, ferrocene derivatives can be transferred across cell membranes (Lai *et al.*, 2019 ▸). Ferrocene is not only an excellent chromophore group, it also performs as an excellent inter­molecular electron and energy-transfer group. The introduction of tri­fluoro­methyl into the compound is conducive to inter­molecular charge transfer, and thus potentially gives the mol­ecule better non-linear optical properties. The *β*-diketonate ligands form stable six-membered metallacycles with transition metals such as Ru, and their terminal groups can be easily modified to change the electronic character of the ligand (Baird *et al.*, 2003 ▸).

**Figure 1:**
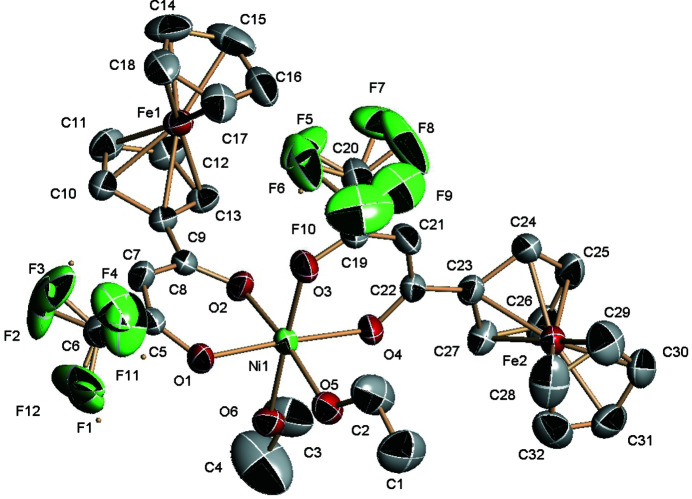
The mol­ecular structure of the title compound, with the atom-numbering scheme. Displacement ellipsoids are drawn at the 30% probability level; H atoms were omitted for clarity.

The mol­ecular structure of the trimetallic title compound is shown in Fig. 1 ▸. The Ni^II^ centre shows an octa­hedral coordination environment built up by the coordination of two chelating *β*-diketonate ligands and two ethanol mol­ecules in a *cis* arrangement. The nickel is placed in general position in the triclinic cell, and the Ni—O coordination bond lengths are in the range 2.003 (2) to 2.149 (2) Å. The *cis* bond angles describing the octa­hedral coordination geometry around Ni^II^ are in the range 85.60 (10) to 93.52 (10)°. The ferrocene moieties substituting the *β*-diketon­ate ligands have the expected geometry, with eclipsed cyclo­penta­diene rings.

**Figure 2:**
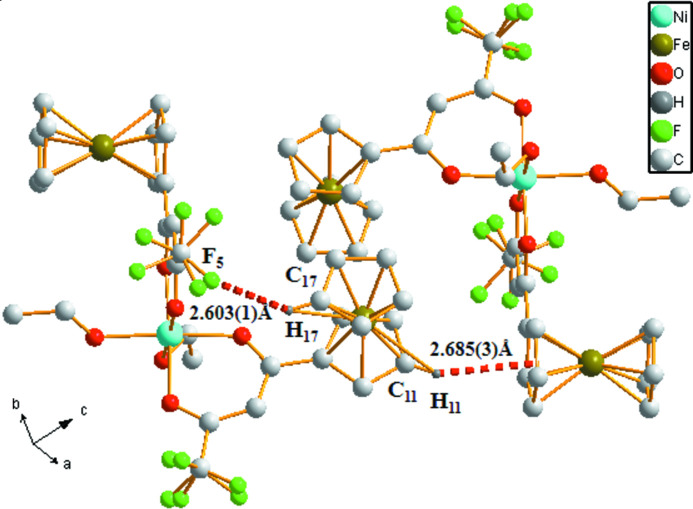
Part of the crystal structure of the title compound, showing some inter­molecular inter­actions.

There are inter­actions between mol­ecules in the crystal structure, through hydrogen bonds involving both coordinated ethanol mol­ecules (Table 1 ▸). Other secondary contacts are C16—H16⋯F5^ii^ and C13—H13⋯C13^iii^. The crystal is further stabilized by C—H⋯π contacts involving cyclo­penta­diene rings of neighbouring mol­ecules (Fig. 2 ▸), giving rise to a three-dimensional architecture.

## Synthesis and crystallization

4,4,4-Tri­fluoro-1-ferrocene­butane-1,3-dione (1.6 mmol, 0.518 g) and tri­ethyl­amine (2.45 mmol, 0.248 g) were dissolved in ethanol (10 ml). Nickel acetate tetra­hydrate (0.5 mmol, 0.122 g) was dissolved in 15 ml of ethanol, added to the previous solution, and stirred at room temperature for 10 min. The mixture was then refluxed for 4 h. After the reaction was complete, the mixture was cooled to room temperature and filtered. The residue was washed twice with 30 ml of ethanol, yielding a red solid (yield: 370 mg, 92%). Single crystals for X-ray analysis were obtained by recrystallization from cyclo­hexane.

## Refinement

Crystal data, data collection and structure refinement details are summarized in Table 2 ▸.

## Supplementary Material

Crystal structure: contains datablock(s) I, global. DOI: 10.1107/S2414314621006933/bh4064sup1.cif

Structure factors: contains datablock(s) I. DOI: 10.1107/S2414314621006933/bh4064Isup2.hkl

CCDC reference: 2012680

Additional supporting information:  crystallographic information; 3D view; checkCIF report

## Figures and Tables

**Table 1:** Hydrogen-bond geometry (Å, °) *Cg* is the centroid of the C23–C27 ring.

*D*—H⋯*A*	*D*—H	H⋯*A*	*D*⋯*A*	*D*—H⋯*A*
O2—H2⋯O3^i^	0.68 (5)	2.24 (5)	2.887 (3)	162 (6)
O3—H3⋯O4^i^	0.78 (4)	2.06 (4)	2.800 (3)	160 (5)
C16—H16⋯F5^ii^	0.93	2.63	3.401 (7)	141
C13—H13⋯C13^iii^	0.93	2.85	3.454 (4)	124
C11—H11⋯*Cg*3^iii^	0.93	2.69	3.520 (5)	150

Symmetry codes: (i) 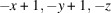 ; (ii) 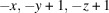 ; (iii) 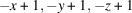 .

**Table 2:** Experimental details

Crystal data	
Chemical formula	[NiFe_2_(C_5_H_5_)(C_9_H_5_F_3_O_2_)_2_(C_2_H_6_O)_2_]
*M*_r_	796.98
Crystal system, space group	Triclinic, *P* 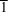
Temperature (K)	296
*a*, *b*, *c* (Å)	10.123 (3), 11.147 (3), 14.869 (4)
α, β, γ (°)	82.741 (3), 77.523 (3), 82.077 (3)
*V* (Å^3^)	1614.5 (8)
*Z*	2
Radiation type	Mo *K*α
μ (mm^−1^)	1.54
Crystal size (mm)	0.05 × 0.03 × 0.02

Data collection	
Diffractometer	Bruker APEXII CCD
Absorption correction	Multi-scan (*SADABS*; Bruker, 2014 ▸)
*T*_min_, *T*_max_	0.484, 0.745
No. of measured, independent and observed [*I* > 2σ(*I*)] reflections	12246, 6266, 5279
*R*_int_	0.029
(sin θ/λ)_max_ (Å^−1^)	0.628

Refinement	
*R*[*F* ^2^ > 2σ(*F* ^2^)], *wR*(*F* ^2^), *S*	0.041, 0.118, 1.05
No. of reflections	6266
No. of parameters	432
H-atom treatment	H atoms treated by a mixture of independent and constrained refinement
Δρ_max_, Δρ_min_ (e Å^−3^)	0.72, −0.53

Computer programs: *APEX2* and *SAINT* (Bruker, 2014 ▸), *SHELXT* (Sheldrick, 2015*a* ▸), *SHELXL* (Sheldrick, 2015*b* ▸), *OLEX2* (Dolomanov *et al.*, 2009 ▸) and *publCIF* (Westrip, 2010 ▸).

## full crystallographic data

### Crystal data

**Table:**

[NiFe_2_(C_14_H_10_F_3_O_2_)_2_(C_2_H_6_O)_2_]	*Z* = 2
*M**_r_* = 796.98	*F*(000) = 812
Triclinic, *P*1	*D*_x_ = 1.639 Mg m^−^^3^
*a* = 10.123 (3) Å	Mo *K*α radiation, λ = 0.71073 Å
*b* = 11.147 (3) Å	Cell parameters from 6629 reflections
*c* = 14.869 (4) Å	θ = 2.3–26.4°
α = 82.741 (3)°	µ = 1.54 mm^−^^1^
β = 77.523 (3)°	*T* = 296 K
γ = 82.077 (3)°	Block, clear reddish black
*V* = 1614.5 (8) Å^3^	0.05 × 0.03 × 0.02 mm

### Data collection

**Table:**

Bruker APEXII CCD diffractometer	5279 reflections with *I* > 2σ(*I*)
Graphite monochromator	*R*_int_ = 0.029
φ and ω scans	θ_max_ = 26.5°, θ_min_ = 1.4°
Absorption correction: multi-scan (SADABS; Bruker, 2014)	*h* = −12→10
*T*_min_ = 0.484, *T*_max_ = 0.745	*k* = −13→13
12246 measured reflections	*l* = −18→18
6266 independent reflections	

### Refinement

**Table:**

Refinement on *F*^2^	Primary atom site location: dual
Least-squares matrix: full	Secondary atom site location: difference Fourier map
*R*[*F*^2^ > 2σ(*F*^2^)] = 0.041	Hydrogen site location: mixed
*wR*(*F*^2^) = 0.118	H atoms treated by a mixture of independent and constrained refinement
*S* = 1.05	*w* = 1/[σ^2^(*F*_o_^2^) + (0.0565*P*)^2^ + 1.4766*P*] where *P* = (*F*_o_^2^ + 2*F*_c_^2^)/3
6266 reflections	(Δ/σ)_max_ = 0.001
432 parameters	Δρ_max_ = 0.72 e Å^−^^3^
0 restraints	Δρ_min_ = −0.53 e Å^−^^3^
0 constraints	

### Special details

**Table:**

Refinement. H atoms for the hydroxy groups of ethanol molecules, H2 and H3, were found in a difference map, and refined with free coordinates and *U*_iso_(H) = 1.5×*U*_eq_(O). Other H atoms were refined using a riding model.

### Fractional atomic coordinates and isotropic or equivalent isotropic displacement parameters (Å^2^)

**Table:**

	*x*	*y*	*z*	*U*_iso_*/*U*_eq_	
Ni1	0.38087 (4)	0.50361 (3)	0.16176 (2)	0.03598 (12)	
Fe1	0.48422 (5)	0.25393 (4)	0.48840 (3)	0.03846 (13)	
Fe2	0.07724 (5)	0.95627 (4)	0.24265 (3)	0.04027 (13)	
O1	0.2129 (2)	0.4287 (2)	0.22821 (16)	0.0489 (6)	
O2	0.2976 (3)	0.5708 (3)	0.04646 (17)	0.0518 (6)	
H2	0.331 (5)	0.546 (5)	0.008 (3)	0.078*	
O3	0.5572 (2)	0.5889 (2)	0.08975 (15)	0.0441 (5)	
H3	0.541 (5)	0.620 (4)	0.043 (3)	0.066*	
O4	0.4540 (2)	0.34975 (19)	0.09715 (14)	0.0427 (5)	
O5	0.4838 (2)	0.44236 (19)	0.26318 (14)	0.0436 (5)	
O6	0.3104 (2)	0.65990 (19)	0.21809 (15)	0.0432 (5)	
C1	0.1286 (5)	0.6571 (6)	−0.0420 (4)	0.0943 (17)	
H1A	0.170001	0.609599	−0.092421	0.141*	
H1B	0.163754	0.734449	−0.052674	0.141*	
H1C	0.031728	0.669441	−0.037491	0.141*	
C2	0.1594 (5)	0.5927 (6)	0.0443 (3)	0.0885 (16)	
H2A	0.113750	0.639933	0.094919	0.106*	
H2B	0.122140	0.515346	0.054685	0.106*	
C3	0.6125 (5)	0.6664 (5)	0.1389 (3)	0.0817 (15)	
H3A	0.551484	0.741338	0.145221	0.098*	
H3B	0.613550	0.626785	0.200760	0.098*	
C4	0.7447 (7)	0.6966 (8)	0.0987 (6)	0.146 (3)	
H4A	0.761644	0.691895	0.033084	0.220*	
H4B	0.810371	0.640575	0.125078	0.220*	
H4C	0.752205	0.777830	0.110499	0.220*	
C5	0.5006 (3)	0.2527 (3)	0.1413 (2)	0.0385 (7)	
C6	0.5175 (4)	0.1406 (3)	0.0896 (2)	0.0512 (8)	
F1	0.5835 (3)	0.1583 (3)	0.00428 (18)	0.0958 (10)	
F2	0.3978 (3)	0.1127 (3)	0.0822 (3)	0.1122 (12)	
F3	0.5745 (5)	0.0419 (2)	0.1280 (3)	0.1271 (15)	
C7	0.5386 (4)	0.2387 (3)	0.2254 (2)	0.0435 (7)	
H7	0.568845	0.160402	0.248038	0.052*	
C8	0.5353 (3)	0.3351 (3)	0.28099 (19)	0.0370 (6)	
C9	0.5958 (3)	0.3085 (3)	0.3632 (2)	0.0381 (6)	
C10	0.6575 (3)	0.1942 (3)	0.3996 (2)	0.0433 (7)	
H10	0.673372	0.121553	0.372267	0.052*	
C11	0.6899 (4)	0.2119 (3)	0.4841 (3)	0.0524 (9)	
H11	0.730821	0.152411	0.522271	0.063*	
C12	0.6501 (4)	0.3347 (3)	0.5015 (2)	0.0508 (8)	
H12	0.660678	0.369869	0.552650	0.061*	
C13	0.5911 (3)	0.3950 (3)	0.4272 (2)	0.0418 (7)	
H13	0.555709	0.476467	0.421291	0.050*	
C14	0.3826 (5)	0.1472 (4)	0.5950 (3)	0.0777 (13)	
H14	0.423580	0.091516	0.635580	0.093*	
C15	0.3386 (5)	0.2722 (5)	0.6059 (3)	0.0835 (16)	
H15	0.344392	0.312447	0.655832	0.100*	
C16	0.2849 (4)	0.3245 (4)	0.5285 (3)	0.0692 (12)	
H16	0.249571	0.405392	0.517801	0.083*	
C17	0.2941 (4)	0.2327 (4)	0.4702 (3)	0.0610 (10)	
H17	0.266182	0.242331	0.413816	0.073*	
C18	0.3528 (4)	0.1233 (4)	0.5117 (3)	0.0601 (10)	
H18	0.368950	0.048229	0.487848	0.072*	
C19	0.1191 (3)	0.4833 (3)	0.2836 (2)	0.0454 (7)	
C20	0.0023 (4)	0.4068 (4)	0.3253 (4)	0.0670 (12)	
F4	−0.0861 (5)	0.4202 (6)	0.2785 (5)	0.237 (4)	
F5	−0.0597 (5)	0.4306 (4)	0.4073 (4)	0.184 (3)	
F6	0.0404 (3)	0.2922 (3)	0.3364 (3)	0.1246 (14)	
C21	0.1045 (4)	0.5994 (3)	0.3088 (2)	0.0508 (8)	
H21	0.026728	0.624167	0.351330	0.061*	
C22	0.2008 (3)	0.6844 (3)	0.2742 (2)	0.0383 (7)	
C23	0.1753 (3)	0.8068 (3)	0.3052 (2)	0.0410 (7)	
C24	0.0596 (4)	0.8595 (3)	0.3692 (2)	0.0507 (8)	
H24	−0.015378	0.820909	0.400587	0.061*	
C25	0.0815 (5)	0.9810 (3)	0.3751 (2)	0.0584 (10)	
H25	0.023114	1.035806	0.411665	0.070*	
C26	0.2055 (4)	1.0048 (3)	0.3169 (3)	0.0568 (9)	
H26	0.242978	1.078023	0.308299	0.068*	
C27	0.2640 (4)	0.8992 (3)	0.2733 (2)	0.0467 (8)	
H27	0.346363	0.891100	0.231045	0.056*	
C28	0.1004 (5)	0.9924 (6)	0.1031 (3)	0.0824 (15)	
H28	0.180584	0.975687	0.060257	0.099*	
C29	0.0596 (5)	1.0991 (5)	0.1446 (3)	0.0760 (13)	
H29	0.107647	1.166592	0.134476	0.091*	
C30	−0.0656 (4)	1.0885 (4)	0.2042 (3)	0.0664 (11)	
H30	−0.115613	1.148052	0.240228	0.080*	
C31	−0.1040 (4)	0.9737 (5)	0.2011 (4)	0.0761 (13)	
H31	−0.182841	0.943148	0.234907	0.091*	
C32	−0.0009 (6)	0.9123 (5)	0.1370 (4)	0.0893 (17)	
H32	0.000225	0.834499	0.120233	0.107*	

### Atomic displacement parameters (Å^2^)

**Table:**

	*U*^11^	*U*^22^	*U*^33^	*U*^12^	*U*^13^	*U*^23^
Ni1	0.0390 (2)	0.0332 (2)	0.0318 (2)	0.00441 (16)	−0.00298 (15)	−0.00509 (15)
Fe1	0.0466 (3)	0.0348 (2)	0.0337 (2)	−0.00839 (19)	−0.00571 (18)	−0.00216 (17)
Fe2	0.0411 (3)	0.0362 (3)	0.0406 (2)	0.00489 (19)	−0.00639 (19)	−0.00622 (18)
O1	0.0500 (14)	0.0356 (12)	0.0544 (13)	−0.0018 (10)	0.0058 (11)	−0.0110 (10)
O2	0.0442 (14)	0.0649 (17)	0.0437 (13)	0.0071 (12)	−0.0112 (11)	−0.0056 (11)
O3	0.0439 (12)	0.0507 (14)	0.0373 (11)	−0.0059 (10)	−0.0072 (10)	−0.0041 (10)
O4	0.0537 (13)	0.0384 (12)	0.0333 (10)	0.0066 (10)	−0.0072 (9)	−0.0092 (9)
O5	0.0629 (15)	0.0313 (11)	0.0348 (10)	0.0052 (10)	−0.0116 (10)	−0.0045 (8)
O6	0.0408 (12)	0.0338 (11)	0.0482 (12)	0.0024 (9)	0.0033 (10)	−0.0063 (9)
C1	0.071 (3)	0.129 (5)	0.083 (3)	−0.005 (3)	−0.037 (3)	0.021 (3)
C2	0.057 (3)	0.136 (5)	0.067 (3)	0.012 (3)	−0.021 (2)	0.003 (3)
C3	0.089 (3)	0.104 (4)	0.062 (3)	−0.040 (3)	−0.011 (2)	−0.024 (2)
C4	0.092 (5)	0.172 (8)	0.192 (8)	−0.063 (5)	0.000 (5)	−0.080 (6)
C5	0.0396 (16)	0.0347 (16)	0.0378 (15)	−0.0031 (13)	0.0002 (12)	−0.0059 (12)
C6	0.064 (2)	0.0387 (18)	0.0518 (19)	−0.0043 (16)	−0.0116 (17)	−0.0084 (15)
F1	0.131 (3)	0.0731 (17)	0.0711 (16)	−0.0160 (16)	0.0277 (16)	−0.0397 (14)
F2	0.089 (2)	0.110 (2)	0.155 (3)	−0.0319 (19)	−0.019 (2)	−0.068 (2)
F3	0.231 (4)	0.0458 (15)	0.126 (3)	0.036 (2)	−0.100 (3)	−0.0345 (16)
C7	0.055 (2)	0.0320 (16)	0.0425 (16)	0.0034 (14)	−0.0115 (14)	−0.0046 (13)
C8	0.0367 (16)	0.0365 (16)	0.0320 (14)	0.0006 (12)	0.0009 (12)	−0.0003 (12)
C9	0.0396 (16)	0.0362 (16)	0.0353 (14)	−0.0041 (13)	−0.0037 (12)	0.0019 (12)
C10	0.0427 (17)	0.0393 (17)	0.0448 (17)	0.0001 (14)	−0.0071 (14)	−0.0001 (13)
C11	0.050 (2)	0.054 (2)	0.055 (2)	−0.0058 (16)	−0.0237 (16)	0.0081 (16)
C12	0.056 (2)	0.053 (2)	0.0499 (19)	−0.0155 (17)	−0.0219 (16)	0.0000 (15)
C13	0.0498 (19)	0.0366 (16)	0.0397 (15)	−0.0125 (14)	−0.0087 (13)	0.0010 (13)
C14	0.097 (4)	0.073 (3)	0.056 (2)	−0.032 (3)	0.002 (2)	0.017 (2)
C15	0.097 (4)	0.091 (4)	0.056 (2)	−0.041 (3)	0.029 (2)	−0.028 (2)
C16	0.055 (2)	0.051 (2)	0.091 (3)	−0.0073 (18)	0.017 (2)	−0.021 (2)
C17	0.045 (2)	0.061 (2)	0.078 (3)	−0.0142 (18)	−0.0081 (18)	−0.007 (2)
C18	0.060 (2)	0.043 (2)	0.076 (3)	−0.0195 (17)	0.0006 (19)	−0.0077 (18)
C19	0.0421 (18)	0.0374 (17)	0.0514 (18)	−0.0022 (14)	−0.0008 (14)	−0.0017 (14)
C20	0.045 (2)	0.049 (2)	0.101 (3)	−0.0060 (17)	0.008 (2)	−0.023 (2)
F4	0.138 (4)	0.291 (7)	0.313 (8)	−0.156 (5)	−0.145 (5)	0.174 (6)
F5	0.183 (4)	0.129 (3)	0.206 (5)	−0.103 (3)	0.126 (4)	−0.079 (3)
F6	0.085 (2)	0.0529 (17)	0.213 (4)	−0.0262 (15)	0.023 (2)	0.000 (2)
C21	0.0459 (19)	0.0430 (19)	0.0542 (19)	−0.0047 (15)	0.0123 (15)	−0.0094 (15)
C22	0.0399 (17)	0.0354 (16)	0.0369 (15)	0.0027 (13)	−0.0067 (13)	−0.0027 (12)
C23	0.0487 (18)	0.0330 (16)	0.0401 (15)	0.0014 (13)	−0.0099 (13)	−0.0041 (12)
C24	0.061 (2)	0.0428 (19)	0.0414 (17)	0.0024 (16)	−0.0008 (15)	−0.0033 (14)
C25	0.088 (3)	0.0423 (19)	0.0433 (18)	0.0107 (19)	−0.0145 (19)	−0.0163 (15)
C26	0.075 (3)	0.043 (2)	0.061 (2)	−0.0039 (18)	−0.032 (2)	−0.0111 (16)
C27	0.0481 (19)	0.0380 (17)	0.0571 (19)	−0.0027 (14)	−0.0192 (15)	−0.0041 (14)
C28	0.071 (3)	0.123 (4)	0.044 (2)	0.024 (3)	−0.015 (2)	−0.006 (2)
C29	0.068 (3)	0.074 (3)	0.070 (3)	0.012 (2)	−0.012 (2)	0.025 (2)
C30	0.053 (2)	0.062 (3)	0.074 (3)	0.0180 (19)	−0.012 (2)	0.001 (2)
C31	0.047 (2)	0.091 (4)	0.091 (3)	−0.001 (2)	−0.021 (2)	−0.008 (3)
C32	0.108 (4)	0.091 (4)	0.089 (3)	0.014 (3)	−0.062 (3)	−0.037 (3)

### Geometric parameters (Å, º)

**Table:**

Ni1—O6	2.003 (2)	C6—F2	1.322 (5)
Ni1—O1	2.006 (2)	C7—C8	1.429 (4)
Ni1—O5	2.012 (2)	C7—H7	0.9300
Ni1—O4	2.043 (2)	C8—C9	1.462 (4)
Ni1—O2	2.082 (2)	C9—C13	1.428 (4)
Ni1—O3	2.149 (2)	C9—C10	1.435 (4)
Fe1—C9	2.026 (3)	C10—C11	1.409 (5)
Fe1—C14	2.035 (4)	C10—H10	0.9300
Fe1—C13	2.038 (3)	C11—C12	1.413 (5)
Fe1—C15	2.040 (4)	C11—H11	0.9300
Fe1—C10	2.041 (3)	C12—C13	1.420 (5)
Fe1—C17	2.050 (4)	C12—H12	0.9300
Fe1—C16	2.051 (4)	C13—H13	0.9300
Fe1—C18	2.054 (4)	C14—C18	1.400 (6)
Fe1—C11	2.059 (4)	C14—C15	1.422 (7)
Fe1—C12	2.067 (3)	C14—H14	0.9300
Fe2—C28	2.031 (4)	C15—C16	1.406 (7)
Fe2—C24	2.032 (3)	C15—H15	0.9300
Fe2—C25	2.033 (3)	C16—C17	1.404 (6)
Fe2—C29	2.035 (4)	C16—H16	0.9300
Fe2—C30	2.036 (4)	C17—C18	1.410 (6)
Fe2—C31	2.036 (4)	C17—H17	0.9300
Fe2—C27	2.039 (3)	C18—H18	0.9300
Fe2—C26	2.039 (4)	C19—C21	1.373 (5)
Fe2—C32	2.043 (4)	C19—C20	1.529 (5)
Fe2—C23	2.044 (3)	C20—F4	1.230 (6)
O1—C19	1.256 (4)	C20—F6	1.283 (5)
O2—C2	1.393 (5)	C20—F5	1.287 (6)
O2—H2	0.68 (5)	C21—C22	1.424 (5)
O3—C3	1.437 (5)	C21—H21	0.9300
O3—H3	0.78 (4)	C22—C23	1.467 (4)
O4—C5	1.280 (4)	C23—C27	1.428 (5)
O5—C8	1.256 (4)	C23—C24	1.446 (5)
O6—C22	1.255 (4)	C24—C25	1.418 (5)
C1—C2	1.461 (6)	C24—H24	0.9300
C1—H1A	0.9600	C25—C26	1.399 (6)
C1—H1B	0.9600	C25—H25	0.9300
C1—H1C	0.9600	C26—C27	1.411 (5)
C2—H2A	0.9700	C26—H26	0.9300
C2—H2B	0.9700	C27—H27	0.9300
C3—C4	1.411 (8)	C28—C29	1.381 (7)
C3—H3A	0.9700	C28—C32	1.420 (8)
C3—H3B	0.9700	C28—H28	0.9300
C4—H4A	0.9600	C29—C30	1.391 (6)
C4—H4B	0.9600	C29—H29	0.9300
C4—H4C	0.9600	C30—C31	1.396 (7)
C5—C7	1.370 (4)	C30—H30	0.9300
C5—C6	1.520 (5)	C31—C32	1.415 (7)
C6—F3	1.295 (5)	C31—H31	0.9300
C6—F1	1.305 (4)	C32—H32	0.9300
			
O6—Ni1—O1	91.01 (9)	F3—C6—C5	115.6 (3)
O6—Ni1—O5	91.94 (9)	F1—C6—C5	112.4 (3)
O1—Ni1—O5	93.52 (10)	F2—C6—C5	110.8 (3)
O6—Ni1—O4	176.77 (8)	C5—C7—C8	125.0 (3)
O1—Ni1—O4	90.86 (9)	C5—C7—H7	117.5
O5—Ni1—O4	90.57 (9)	C8—C7—H7	117.5
O6—Ni1—O2	89.89 (10)	O5—C8—C7	123.8 (3)
O1—Ni1—O2	93.30 (11)	O5—C8—C9	117.7 (3)
O5—Ni1—O2	172.91 (10)	C7—C8—C9	118.5 (3)
O4—Ni1—O2	87.38 (10)	C13—C9—C10	107.5 (3)
O6—Ni1—O3	87.69 (9)	C13—C9—C8	123.7 (3)
O1—Ni1—O3	178.29 (9)	C10—C9—C8	128.6 (3)
O5—Ni1—O3	87.64 (10)	C13—C9—Fe1	69.88 (17)
O4—Ni1—O3	90.39 (9)	C10—C9—Fe1	69.89 (17)
O2—Ni1—O3	85.60 (10)	C8—C9—Fe1	121.4 (2)
C9—Fe1—C14	161.24 (17)	C11—C10—C9	107.6 (3)
C9—Fe1—C13	41.13 (13)	C11—C10—Fe1	70.6 (2)
C14—Fe1—C13	156.45 (17)	C9—C10—Fe1	68.78 (17)
C9—Fe1—C15	156.13 (19)	C11—C10—H10	126.2
C14—Fe1—C15	40.8 (2)	C9—C10—H10	126.2
C13—Fe1—C15	120.84 (17)	Fe1—C10—H10	126.0
C9—Fe1—C10	41.32 (12)	C10—C11—C12	109.0 (3)
C14—Fe1—C10	124.28 (18)	C10—C11—Fe1	69.22 (19)
C13—Fe1—C10	68.94 (14)	C12—C11—Fe1	70.3 (2)
C15—Fe1—C10	161.3 (2)	C10—C11—H11	125.5
C9—Fe1—C17	107.79 (15)	C12—C11—H11	125.5
C14—Fe1—C17	67.8 (2)	Fe1—C11—H11	126.6
C13—Fe1—C17	124.62 (15)	C11—C12—C13	107.9 (3)
C15—Fe1—C17	67.4 (2)	C11—C12—Fe1	69.7 (2)
C10—Fe1—C17	122.27 (16)	C13—C12—Fe1	68.68 (19)
C9—Fe1—C16	120.97 (17)	C11—C12—H12	126.0
C14—Fe1—C16	68.2 (2)	C13—C12—H12	126.0
C13—Fe1—C16	107.32 (16)	Fe1—C12—H12	127.2
C15—Fe1—C16	40.2 (2)	C12—C13—C9	108.0 (3)
C10—Fe1—C16	157.09 (17)	C12—C13—Fe1	70.86 (19)
C17—Fe1—C16	40.04 (17)	C9—C13—Fe1	68.99 (17)
C9—Fe1—C18	124.92 (15)	C12—C13—H13	126.0
C14—Fe1—C18	40.06 (18)	C9—C13—H13	126.0
C13—Fe1—C18	161.60 (16)	Fe1—C13—H13	125.7
C15—Fe1—C18	67.51 (18)	C18—C14—C15	107.4 (4)
C10—Fe1—C18	108.46 (15)	C18—C14—Fe1	70.7 (2)
C17—Fe1—C18	40.20 (17)	C15—C14—Fe1	69.8 (2)
C16—Fe1—C18	67.55 (17)	C18—C14—H14	126.3
C9—Fe1—C11	68.35 (13)	C15—C14—H14	126.3
C14—Fe1—C11	108.35 (19)	Fe1—C14—H14	124.8
C13—Fe1—C11	67.97 (14)	C16—C15—C14	108.3 (4)
C15—Fe1—C11	125.2 (2)	C16—C15—Fe1	70.3 (2)
C10—Fe1—C11	40.19 (14)	C14—C15—Fe1	69.4 (2)
C17—Fe1—C11	157.55 (16)	C16—C15—H15	125.9
C16—Fe1—C11	161.17 (18)	C14—C15—H15	125.9
C18—Fe1—C11	122.58 (16)	Fe1—C15—H15	126.0
C9—Fe1—C12	68.47 (14)	C17—C16—C15	107.7 (4)
C14—Fe1—C12	121.71 (18)	C17—C16—Fe1	69.9 (2)
C13—Fe1—C12	40.46 (13)	C15—C16—Fe1	69.5 (3)
C15—Fe1—C12	108.11 (18)	C17—C16—H16	126.2
C10—Fe1—C12	68.00 (15)	C15—C16—H16	126.2
C17—Fe1—C12	161.10 (16)	Fe1—C16—H16	126.0
C16—Fe1—C12	124.70 (17)	C16—C17—C18	108.4 (4)
C18—Fe1—C12	156.99 (16)	C16—C17—Fe1	70.0 (2)
C11—Fe1—C12	40.04 (15)	C18—C17—Fe1	70.0 (2)
C28—Fe2—C24	159.6 (2)	C16—C17—H17	125.8
C28—Fe2—C25	158.6 (2)	C18—C17—H17	125.8
C24—Fe2—C25	40.85 (15)	Fe1—C17—H17	125.7
C28—Fe2—C29	39.7 (2)	C14—C18—C17	108.3 (4)
C24—Fe2—C29	159.77 (18)	C14—C18—Fe1	69.3 (2)
C25—Fe2—C29	121.8 (2)	C17—C18—Fe1	69.7 (2)
C28—Fe2—C30	67.06 (18)	C14—C18—H18	125.9
C24—Fe2—C30	124.86 (16)	C17—C18—H18	125.9
C25—Fe2—C30	105.46 (17)	Fe1—C18—H18	126.7
C29—Fe2—C30	39.96 (17)	O1—C19—C21	130.3 (3)
C28—Fe2—C31	68.1 (2)	O1—C19—C20	112.9 (3)
C24—Fe2—C31	109.31 (19)	C21—C19—C20	116.7 (3)
C25—Fe2—C31	120.04 (19)	F4—C20—F6	106.9 (5)
C29—Fe2—C31	67.7 (2)	F4—C20—F5	106.0 (5)
C30—Fe2—C31	40.10 (19)	F6—C20—F5	102.9 (5)
C28—Fe2—C27	108.43 (18)	F4—C20—C19	112.6 (4)
C24—Fe2—C27	69.10 (15)	F6—C20—C19	113.8 (3)
C25—Fe2—C27	68.20 (16)	F5—C20—C19	113.9 (4)
C29—Fe2—C27	118.64 (17)	C19—C21—C22	124.3 (3)
C30—Fe2—C27	152.05 (17)	C19—C21—H21	117.9
C31—Fe2—C27	166.41 (18)	C22—C21—H21	117.9
C28—Fe2—C26	123.6 (2)	O6—C22—C21	123.2 (3)
C24—Fe2—C26	68.53 (16)	O6—C22—C23	116.8 (3)
C25—Fe2—C26	40.20 (17)	C21—C22—C23	120.0 (3)
C29—Fe2—C26	104.4 (2)	C27—C23—C24	106.9 (3)
C30—Fe2—C26	117.13 (18)	C27—C23—C22	124.3 (3)
C31—Fe2—C26	152.59 (19)	C24—C23—C22	128.8 (3)
C27—Fe2—C26	40.49 (15)	C27—C23—Fe2	69.32 (18)
C28—Fe2—C32	40.8 (2)	C24—C23—Fe2	68.78 (18)
C24—Fe2—C32	124.0 (2)	C22—C23—Fe2	125.5 (2)
C25—Fe2—C32	157.0 (2)	C25—C24—C23	107.3 (3)
C29—Fe2—C32	67.8 (2)	C25—C24—Fe2	69.6 (2)
C30—Fe2—C32	67.5 (2)	C23—C24—Fe2	69.66 (18)
C31—Fe2—C32	40.6 (2)	C25—C24—H24	126.3
C27—Fe2—C32	128.29 (19)	C23—C24—H24	126.3
C26—Fe2—C32	162.7 (2)	Fe2—C24—H24	126.0
C28—Fe2—C23	123.33 (17)	C26—C25—C24	108.9 (3)
C24—Fe2—C23	41.56 (13)	C26—C25—Fe2	70.2 (2)
C25—Fe2—C23	68.93 (13)	C24—C25—Fe2	69.55 (19)
C29—Fe2—C23	155.42 (16)	C26—C25—H25	125.6
C30—Fe2—C23	164.37 (16)	C24—C25—H25	125.6
C31—Fe2—C23	129.01 (18)	Fe2—C25—H25	126.3
C27—Fe2—C23	40.96 (13)	C25—C26—C27	108.6 (3)
C26—Fe2—C23	68.63 (14)	C25—C26—Fe2	69.6 (2)
C32—Fe2—C23	111.63 (18)	C27—C26—Fe2	69.7 (2)
C19—O1—Ni1	122.9 (2)	C25—C26—H26	125.7
C2—O2—Ni1	126.2 (2)	C27—C26—H26	125.7
C2—O2—H2	109 (5)	Fe2—C26—H26	126.5
Ni1—O2—H2	113 (5)	C26—C27—C23	108.3 (3)
C3—O3—Ni1	117.9 (2)	C26—C27—Fe2	69.8 (2)
C3—O3—H3	114 (3)	C23—C27—Fe2	69.72 (19)
Ni1—O3—H3	107 (3)	C26—C27—H27	125.8
C5—O4—Ni1	121.03 (19)	C23—C27—H27	125.8
C8—O5—Ni1	126.9 (2)	Fe2—C27—H27	126.2
C22—O6—Ni1	128.2 (2)	C29—C28—C32	108.5 (4)
C2—C1—H1A	109.5	C29—C28—Fe2	70.3 (2)
C2—C1—H1B	109.5	C32—C28—Fe2	70.1 (3)
H1A—C1—H1B	109.5	C29—C28—H28	125.8
C2—C1—H1C	109.5	C32—C28—H28	125.8
H1A—C1—H1C	109.5	Fe2—C28—H28	125.5
H1B—C1—H1C	109.5	C28—C29—C30	108.3 (5)
O2—C2—C1	114.7 (4)	C28—C29—Fe2	70.0 (3)
O2—C2—H2A	108.6	C30—C29—Fe2	70.1 (2)
C1—C2—H2A	108.6	C28—C29—H29	125.9
O2—C2—H2B	108.6	C30—C29—H29	125.9
C1—C2—H2B	108.6	Fe2—C29—H29	125.6
H2A—C2—H2B	107.6	C29—C30—C31	108.9 (4)
C4—C3—O3	116.2 (5)	C29—C30—Fe2	70.0 (2)
C4—C3—H3A	108.2	C31—C30—Fe2	70.0 (2)
O3—C3—H3A	108.2	C29—C30—H30	125.5
C4—C3—H3B	108.2	C31—C30—H30	125.5
O3—C3—H3B	108.2	Fe2—C30—H30	126.1
H3A—C3—H3B	107.4	C30—C31—C32	107.5 (5)
C3—C4—H4A	109.5	C30—C31—Fe2	69.9 (2)
C3—C4—H4B	109.5	C32—C31—Fe2	70.0 (3)
H4A—C4—H4B	109.5	C30—C31—H31	126.2
C3—C4—H4C	109.5	C32—C31—H31	126.2
H4A—C4—H4C	109.5	Fe2—C31—H31	125.4
H4B—C4—H4C	109.5	C31—C32—C28	106.8 (5)
O4—C5—C7	129.0 (3)	C31—C32—Fe2	69.4 (3)
O4—C5—C6	113.3 (3)	C28—C32—Fe2	69.2 (3)
C7—C5—C6	117.7 (3)	C31—C32—H32	126.6
F3—C6—F1	108.2 (4)	C28—C32—H32	126.6
F3—C6—F2	104.6 (4)	Fe2—C32—H32	126.4
F1—C6—F2	104.4 (3)		
			
Ni1—O2—C2—C1	171.1 (4)	C16—C17—C18—Fe1	−59.8 (3)
Ni1—O3—C3—C4	165.1 (5)	Ni1—O1—C19—C21	2.5 (6)
Ni1—O4—C5—C7	−15.8 (5)	Ni1—O1—C19—C20	−179.9 (3)
Ni1—O4—C5—C6	165.8 (2)	O1—C19—C20—F4	−89.4 (6)
O4—C5—C6—F3	174.7 (4)	C21—C19—C20—F4	88.5 (6)
C7—C5—C6—F3	−3.9 (5)	O1—C19—C20—F6	32.5 (6)
O4—C5—C6—F1	49.8 (4)	C21—C19—C20—F6	−149.6 (4)
C7—C5—C6—F1	−128.8 (4)	O1—C19—C20—F5	149.9 (5)
O4—C5—C6—F2	−66.5 (4)	C21—C19—C20—F5	−32.2 (6)
C7—C5—C6—F2	114.9 (4)	O1—C19—C21—C22	−0.2 (7)
O4—C5—C7—C8	−1.3 (6)	C20—C19—C21—C22	−177.7 (4)
C6—C5—C7—C8	177.0 (3)	Ni1—O6—C22—C21	0.5 (5)
Ni1—O5—C8—C7	5.5 (4)	Ni1—O6—C22—C23	179.4 (2)
Ni1—O5—C8—C9	−174.8 (2)	C19—C21—C22—O6	−1.5 (6)
C5—C7—C8—O5	7.6 (5)	C19—C21—C22—C23	179.7 (3)
C5—C7—C8—C9	−172.0 (3)	O6—C22—C23—C27	2.3 (5)
O5—C8—C9—C13	4.5 (4)	C21—C22—C23—C27	−178.7 (3)
C7—C8—C9—C13	−175.9 (3)	O6—C22—C23—C24	−179.6 (3)
O5—C8—C9—C10	178.4 (3)	C21—C22—C23—C24	−0.7 (5)
C7—C8—C9—C10	−1.9 (5)	O6—C22—C23—Fe2	90.1 (3)
O5—C8—C9—Fe1	90.0 (3)	C21—C22—C23—Fe2	−90.9 (4)
C7—C8—C9—Fe1	−90.3 (3)	C27—C23—C24—C25	−0.6 (4)
C13—C9—C10—C11	−0.2 (4)	C22—C23—C24—C25	−178.9 (3)
C8—C9—C10—C11	−174.9 (3)	Fe2—C23—C24—C25	−59.7 (2)
Fe1—C9—C10—C11	−60.2 (2)	C27—C23—C24—Fe2	59.1 (2)
C13—C9—C10—Fe1	60.0 (2)	C22—C23—C24—Fe2	−119.2 (3)
C8—C9—C10—Fe1	−114.7 (3)	C23—C24—C25—C26	0.4 (4)
C9—C10—C11—C12	−0.1 (4)	Fe2—C24—C25—C26	−59.3 (3)
Fe1—C10—C11—C12	−59.2 (3)	C23—C24—C25—Fe2	59.7 (2)
C9—C10—C11—Fe1	59.1 (2)	C24—C25—C26—C27	−0.1 (4)
C10—C11—C12—C13	0.4 (4)	Fe2—C25—C26—C27	−59.0 (2)
Fe1—C11—C12—C13	−58.2 (2)	C24—C25—C26—Fe2	58.9 (3)
C10—C11—C12—Fe1	58.5 (2)	C25—C26—C27—C23	−0.3 (4)
C11—C12—C13—C9	−0.5 (4)	Fe2—C26—C27—C23	−59.3 (2)
Fe1—C12—C13—C9	−59.2 (2)	C25—C26—C27—Fe2	59.0 (3)
C11—C12—C13—Fe1	58.8 (3)	C24—C23—C27—C26	0.5 (4)
C10—C9—C13—C12	0.4 (4)	C22—C23—C27—C26	179.0 (3)
C8—C9—C13—C12	175.4 (3)	Fe2—C23—C27—C26	59.3 (2)
Fe1—C9—C13—C12	60.4 (2)	C24—C23—C27—Fe2	−58.8 (2)
C10—C9—C13—Fe1	−60.0 (2)	C22—C23—C27—Fe2	119.7 (3)
C8—C9—C13—Fe1	115.0 (3)	C32—C28—C29—C30	0.0 (5)
C18—C14—C15—C16	−1.2 (5)	Fe2—C28—C29—C30	−59.9 (3)
Fe1—C14—C15—C16	59.8 (3)	C32—C28—C29—Fe2	59.9 (3)
C18—C14—C15—Fe1	−61.0 (3)	C28—C29—C30—C31	0.5 (5)
C14—C15—C16—C17	0.6 (5)	Fe2—C29—C30—C31	−59.3 (3)
Fe1—C15—C16—C17	59.7 (3)	C28—C29—C30—Fe2	59.8 (3)
C14—C15—C16—Fe1	−59.2 (3)	C29—C30—C31—C32	−0.8 (5)
C15—C16—C17—C18	0.3 (5)	Fe2—C30—C31—C32	−60.1 (3)
Fe1—C16—C17—C18	59.8 (3)	C29—C30—C31—Fe2	59.3 (3)
C15—C16—C17—Fe1	−59.5 (3)	C30—C31—C32—C28	0.8 (5)
C15—C14—C18—C17	1.4 (5)	Fe2—C31—C32—C28	−59.3 (3)
Fe1—C14—C18—C17	−59.0 (3)	C30—C31—C32—Fe2	60.1 (3)
C15—C14—C18—Fe1	60.4 (3)	C29—C28—C32—C31	−0.5 (5)
C16—C17—C18—C14	−1.1 (5)	Fe2—C28—C32—C31	59.5 (3)
Fe1—C17—C18—C14	58.7 (3)	C29—C28—C32—Fe2	−60.0 (3)

### Hydrogen-bond geometry (Å, º)

*Cg* is the centroid of the C23–C27 ring.

**Table:**

*D*—H···*A*	*D*—H	H···*A*	*D*···*A*	*D*—H···*A*
O2—H2···O3^i^	0.68 (5)	2.24 (5)	2.887 (3)	162 (6)
O3—H3···O4^i^	0.78 (4)	2.06 (4)	2.800 (3)	160 (5)
C3—H3*B*···O5	0.97	2.56	3.168 (6)	121
C16—H16···F5^ii^	0.93	2.63	3.401 (7)	141
C13—H13···C13^iii^	0.93	2.85	3.454 (4)	124
C11—H11···*Cg*3^iii^	0.93	2.69	3.520 (5)	150

Symmetry codes: (i) −*x*+1, −*y*+1, −*z*; (ii) −*x*, −*y*+1, −*z*+1; (iii) −*x*+1, −*y*+1, −*z*+1.
